# Barriers and Facilitators in the Junior-to-Senior Transition in Male Football—A Scoping Review

**DOI:** 10.3390/sports13120440

**Published:** 2025-12-05

**Authors:** João Tomás, Duarte Araújo, Diogo Martinho, João Ribeiro, Honorato Sousa, Adam Field, Hugo Sarmento

**Affiliations:** 1 Universidade de Coimbra, CIPER, FCDEFUC, 3004-531 Coimbra, Portugal; joaotomas9@gmail.com (J.T.); dvmartinho92@hotmail.com (D.M.); 2 CIPER, Faculdade de Motricidade Humana, Universidade de Lisboa, 1649-004 Lisboa, Portugal; daraujo@fmh.ulisboa.pt; 3 Research Center in Sports Sciences, Health Sciences and Human Development, CIDESD, Universidade da Maia, 4475-690 Maia, Portugal; jamribeiro@icloud.com; 4 Gabinete de Otimização Desportiva, GOD, Sporting Clube de Braga, 4700-048 Braga, Portugal; 5 Department of Physical Education and Sport, University of Madeira, 9000-072 Funchal, Portugal; honoratosousa@hotmail.com; 6 Department of Sport and Exercise Science, Institute of Sport, Manchester Metropolitan University, Manchester M15 6BX, UK; a.field@mmu.ac.uk

**Keywords:** expertise, football, football player, performance, training

## Abstract

Background: Despite many young players showing strong potential, only a small fraction succeeds in the critical transition from youth to elite senior football. This scoping review synthesizes research on the junior-to-senior transition in men’s football, identifying main topics related with barriers and facilitators in the transition. Methods: Searches were performed in four databases (PubMed, Scopus, SPORTDiscus, and Web of Science) according to the Preferred Reporting Items for Systematic Reviews and Meta-Analyses (PRISMA, 2020) guidelines, using the following keywords: “football*” OR football AND talent* OR “talent identification” OR “talent development” OR expert* OR gift* AND “junior-to-senior” OR “transition career” or “athlete career transition” OR “transition phase”. Original articles in English focused on the junior-to-senior process in male footballers were included. Results: From 5307 titles, 35 studies met eligibility criteria. The most examined themes were psychosocial factors, including social support, stressors, and resilience. The reviewed studies identified organizational structure and effective club communication as facilitators and emphasized the importance of physical attributes to meet senior-level demands. Conclusions: Overall, the junior-to-senior transition is multifaceted, shaped by psychosocial, organizational, and physical factors. Despite robust research, gaps remain; future longitudinal and interdisciplinary studies should inform evidence-based strategies for optimizing player development and retention.

## 1. Introduction

It is widely acknowledged that the talent development processes in football is both complex and multidimensional [1]. Studies demonstrated that early success in youth football does not necessarily predict achievement at the senior level, highlighting the complex and non-linear nature of talent development pathways [2,3]. To improve progression from elite junior to elite senior levels of sport, a clear understanding of talent development is necessary.

Entry into a professional first team remains the primary benchmark for evaluating a successful athletic career in football [4,5]. A comprehensive understanding of athletic careers requires exploring the interplay between athletes’ multiple life domains (e.g., sport, education, employment) and the developmental trajectories they navigate as talented youth progress through their careers [6]. Effectively managing transitions within and beyond sport enhances an athlete’s likelihood of sustaining a long and successful sporting career and facilitates a smoother adjustment to retirement. Conversely, difficulties in coping with transitional phases are frequently associated with negative outcomes, including premature dropout from sport [7]. Within an ecological dynamics’ framework, “barriers” are constraints in the performer–environment relationship that limit young players’ capacity to adapt to senior-level demands, while “facilitators” denote supportive constraints and resources that enhance perception–action coupling, learning opportunities, and functional adaptation during the junior-to-senior transition [8,9].

Within the talent development process, the primary objective of football academies is to identify and nurture young talented players, an endeavour typically associated with achieving sporting success and generating financial returns for the club [10]. Nevertheless, statistics show that fewer than 10% of junior football players progress to the professional level, meaning that over 90% do not achieve professional status [5,11,12]. These players typically either leave the academies and are replaced by emerging talent, continue participating at a recreational level, or withdraw from sport entirely [13,14].

To reach professional status, football players must successfully navigate the junior-to-senior transition (JST), a critical phase marked by progression from junior (under-19) to senior (open age) competitions, which is especially important for those aspiring to elite levels in the sport. Research consistently identifies the JST as the most critical and challenging phase within an athlete’s career-long talent development in football, typically lasting between one and four years [15,16], and is considered as the most career-defining transition [17]. The JST is well known for its high dropout rate, and about 80% of athletes experience the JST as a crisis. Difficulties arise when young athletes lack the personal and contextual resources to manage increased performance demands, emotional pressures, and shifting social expectations. Factors such as weak coping skills, marginalization of athletic identity, insufficient institutional support, and negative sociocultural influences heighten vulnerability. Emotional barriers, unexpected events like injuries, and the absence of positive role models further hinder adaptation. Without adequate guidance and intervention, these challenges reinforce a downward spiral of stagnation and disengagement [18].

Stambulova and Samuel [19] defined “transitions as turning phases in athletes’ career development associated with a set of specific demands that athletes have to cope with in order to continue successfully in sport and/or the spheres of their life’s” (p. 119). Early work on talent development, such as Bloom’s [20] work, identified distinct stages through which talented individuals progress across domains, including, science, art, and sport. This talent development pathway comprises three stages: (1) the initiation stage where young athletes are introduced to organized sports and during which they are identified as talented athletes; (2) the development stage during which athletes become more dedicated to their sport and where the amount of training and level of specialization is increased; (3) the mastery or perfection stage in which athletes reach their highest level of athletic proficiency.

Several theoretical frameworks have shaped research on talent identification and development. Early work, notably on deliberate practice [21], laid the foundation, but later models emphasized the importance of transitions in athlete development. Key frameworks include: (1) the Athletic Career Transition Model [15,22], which views transition as coping with specific demands; (2) the Holistic Athletic Career Model [23], linking athletic, psychological, psychosocial, academic/vocational, financial domains across a career; (3) the Athletic Talent Development Environment Model ATDE: [24,25,26] which situates the junior-to-senior transition within a dynamic ecological system. Inspired by Bronfenbrenner’s [27] bio-ecological theory the ATDE highlights the layered environment influencing development, from immediate micro-level settings and their interrelations, through macro-level contexts, to the organizational culture of clubs, which collectively shape the effectiveness of talent development [28].

From a talent development perspective, a normative (predictable) from junior elite to senior elite would be ideal; however, the volatile nature of professional football often makes this transition unpredictable and stressful for players [29,30,31].

Current research on the junior-to-senior transition in football remains limited and predominantly cross-sectional, with most studies focusing on youth stages rather than systematically tracking outcomes at the senior level [32]. The existing literature underscores the multifaceted nature of this transition, which involves increased performance demands, intensified psychological and physical stressors, and substantial shifts in social and organizational contexts. Furthermore, a range of additional factors, including coaching approaches, competition intensity, injury management, psychological resilience, and the availability of social and institutional support, physical and maturational factors may also play a decisive role in shaping young players’ adaptation during this critical developmental stage [17,33,34,35].

The aim of this review was to organize and synthesize the available scientific literature on the male junior-to-senior transition in football, identifying key research topics and the main barriers and facilitators in this transition.

## 2. Materials and Methods

The present review was conducted following the Preferred Reporting Items for Systematic Reviews and Meta- Analyses extension for Scoping Reviews (PRISMA-ScR) [36]. The protocol was registered by an expert element on the Open Science Framework (https://doi.org/10.17605/OSF.IO/JBZXR, accessed on 26 June 2025).

### 2.1. Eligibility Criteria

The present review included studies that: (1) centered their attention in male football junior-to-senior transition; (2) contained relevant information on the junior-to-senior transition process; (3) examined changes over time and offered insights into development within the review’s context; (4) were original studies written in English. The studies were excluded if they: (1) included data from female athletes; (2) do not include relevant information concerning the topic under study; (3) were written in language others than English; (4) were grey literature.

### 2.2. Information Sources, Search and Selection Process

Four databases were searched (PubMed, Scopus, SPORTDiscus, and Web of Science) for published articles until 24 March 2024 (date of the search) using the following search terms: (“football*” OR soccer) AND (talent* OR “talent identification” OR “talent development” OR expert* OR gift*) AND (“junior-to-senior” OR “transition career” or “athlete career transition” OR “transition phase”). Subsequently, the references were exported to a citation management software (EndNoteTM 21.0, ClarivateTM), that was used also for the screening process. Duplicates were automatically removed and manually checked to ensure that duplicates were removed with precision. Two independent authors (JT and HS) examined the manuscripts for eligibility based on the title, abstract and full text review. Where discrepancies occurred, a third author (DVM) was consulted and solved the disagreements by consensus.

### 2.3. Quality of the Studies, Data Extraction and Synthesis

The overall methodological quality of the studies was assessed using two tools: (1) the 21-item Critical Review Form by Letts et al. [37] for qualitative studies; (2) the 16 item critical review form for quantitative studies by Law et al. [38].

Each qualitative article was subjected to an objective assessment to determine whether it contained the 21 critical components: objective (item 1), literature reviewed (item 2), study design (items 3, 4 and 5), sampling (items 6, 7, 8 and 9), data collection (descriptive clarity: items 10, 11 and 12; procedural rigor: item 13), data analyses (analytical rigor: items 14 and 15; auditability: items 16 and 17; theoretical connections: item 18), overall rigor (item 19) and conclusion/implications (items 20 and 21). On the other hand, quantitative studies were assessed to determine whether they included the16 items: objective (item 1), relevance of background literature (item 2), appropriateness of the study design (item 3), sample included (items 4 and 5), informed consent procedure (item 6), outcome measures (item 7), validity of measures (item 8), significance of results (item 10), analysis (item 11), clinical importance (item 12), description of drop-outs (item 13), conclusion (item 14), practical implications (item 15) and limitations (item 16). Item 9 (details of the intervention procedure) was not applicable because none of the studies included interventions.

The outcomes per item were 1 (meets criteria), 0 (does not meet the criteria fully), or NA (not applicable). A final score expressed as a percentage was calculated for each study by following the scoring guidelines of Faber et al. [39]. This final score corresponded to the sum of every score in each article divided by the total number of items scored for that specific research design (i.e., 16 or 21 items). As in previous studies (see, for example, Sarmento et al. [40]) we adopted the classifications of Faber, Bustin, Oosterveld, Elferink-Gemser and Nijhuis-Van der Sanden [39] and Te Wierike et al. [41] and classified the articles as: (1) low methodological quality—with a score ≤50%; (2) good methodological quality—score between 51 and 75%; and (3) excellent methodological quality—with a score > 75%.

The methodological quality of the studies was evaluated independently by two authors (JT and DM). A third author (HS) was consulted to solve the cases of disagreement between the two authors.

A data extraction sheet (from Cochrane Consumers and Communication Review Group’s data extraction template [42], was customized to this review’s study inclusion requirements and then pilot tested on ten randomly selected studies. Data were extracting according to the Participants/Exposure/Outcomes framework developed to build the search question. Based on each study’s aim, two authors (JT and HS) independently extracted the main data.

## 3. Results

### 3.1. Search, Selection and Inclusion Publication

**Figure 1:**
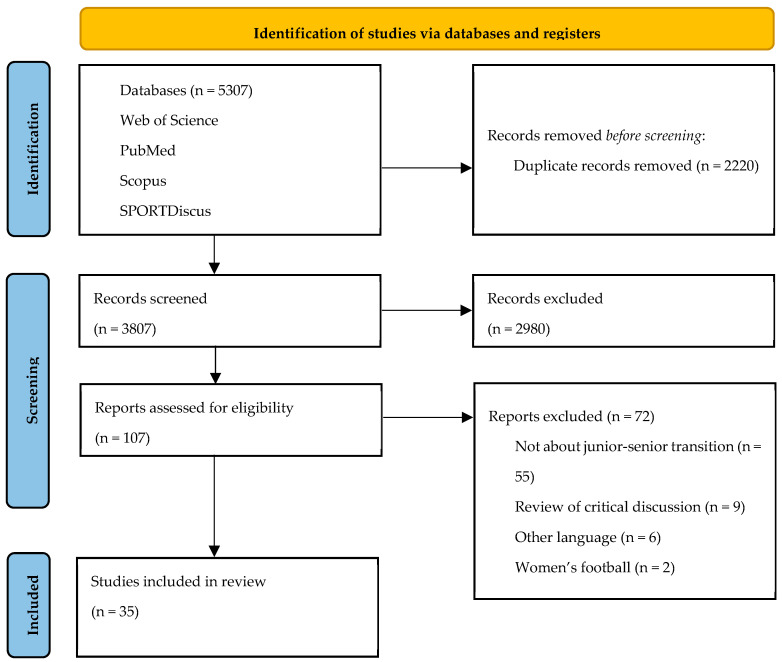
PRISMA flow chart of the studies included in the present review.

The search performed on databases identified 5307 manuscripts. Duplicates were removed (2220 studies) and the title and abstract of the 3087 remaining papers were screened according to the eligibility criteria. Subsequently, 2980 papers were deleted, and 107 records were examined in full text. Of these, 72 studies did not meet the inclusion criteria and were removed for the following reasons: (1) the paper did not present relevant information concerning junior-to-senior transition (*n* = 55); (2) the manuscripts are not an original paper (*n* = 9); (3) six articles were not written in English; (4) two articles were related to women’s football. Finally, 35 manuscripts were included in the present review, and they were retrieved for the analysis (Figure 1).

### 3.2. Quality of Studies

Concerning the quality of studies, the most noteworthy results were that: (1) the mean score for the 21 selected quantitative studies was 89.17%; (2) the mean score for the fourteen selected qualitative studies was 88.47%; (3) two publications achieved the maximum score of 100%; (4) no publication scored below 75%; (5) fourteen studies scored between 76 and 89%; and (6) 17 publications achieved an overall rating of >90%. All studies included in present review exceeded the minimum quality threshold, and therefore quality scores did not influence the synthesis of results. The versions of the Critical Review Forms used in this study are shown in Electronic Supplementary Material Tables S1 and S2.

### 3.3. General Description of Studies

**Figure 2:**
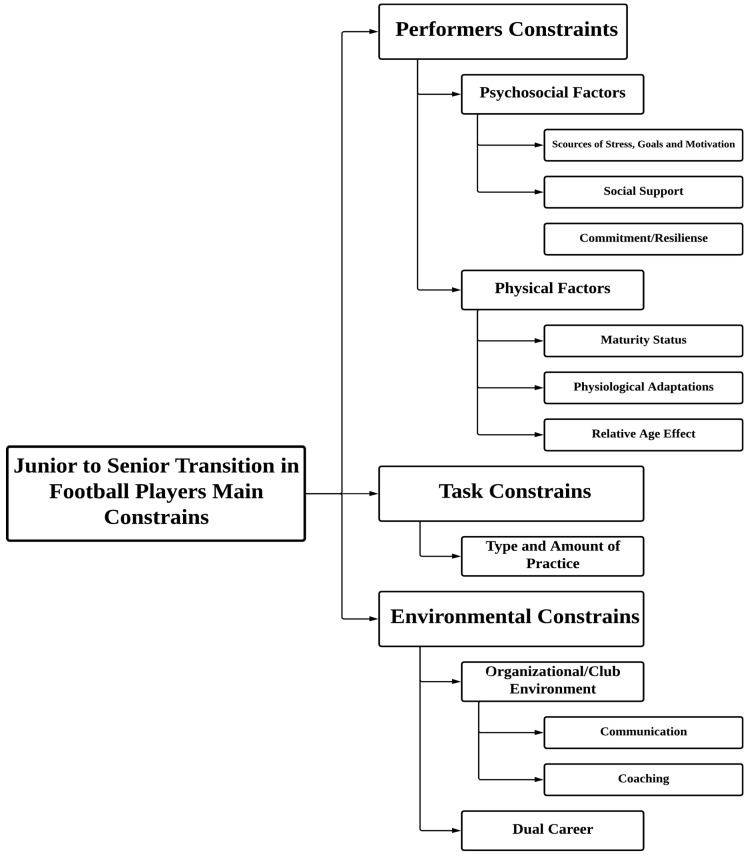
Scopes about junior-to-senior transition in football.

The ecological dynamics framework posits that behaviour can only be understood at the level of the performer–environment relationship, where interactions are shaped by a constellation of personal, task, and environmental constraints operating across multiple, interdependent timescales [43]. Based on this theoretical rationale, after careful analysis, and following a similar strategy of previous reviews on this topic [40,44], it was decided that the most appropriate way to present the results would be to categorise them according to the major research topics that emerged from the analysis (Figure 2): (1) performers constrains: psychosocial and physical factors; (2) task constrains; (3) environmental constrains: organizational/club environment and dual career.

The sample characteristics (i.e., country, number of participants, sport, and age) are summarized in Table 1. Fourteen manuscripts (40%) included studies related to psychological aspects, seven (20%) included physical characteristics, and fourteen (40%) organizational, communicational, dual career, training characteristics and other topics. The studies included in this review involved players form different countries in Europe, North America, and Asia, covering different contexts, in which their analysis proved to be relevant, contributing to a more comprehensive and detailed understanding of the subject in question. The 35 manuscripts analysed in this review include studies carried out between 2005 and 2023. However, there has been a significant increase in the number of publications over the last decade.

Based on an ecological dynamics theoretical approach, we argue that talent should be considered as a dynamically varying relationship shaped by the constraints imposed by the physical and social environments, the tasks experienced and the personal resources of a player [45]. The context of modern football is characterised by repeated evaluation of footballers’ potential to succeed at the elite, adult level. Studies were organized into three main topics: performer constrains (i.e., studies that combined psychosocial constrains and physical constrains) (Table 1); task constrains (Table 2); and environmental constrains (organization/club environment and dual careers) (Table 3).

#### 3.3.1. Performers Constraints

##### Psychosocial Factors

The psychosocial influences were examined in 14 studies (Table 1). From those, five studies mentioned the importance of social support that can impact on young players success [46,47,48,49,50]. Studies that examined commitment or resilience variables (considering the Athlete’s thoughts or experiences were topics covered in three studies [51,52,53,54]). Goal-setting, motivation and the importance of strategically planning their careers in order to reach their ultimate goals were represented in one study [53]. Other topics covered focused on sources of stress that influence high and consistent performance [54,55,56,57,58,59].

**Table 1:** Studies about performers’ constraints in the junior-to-senior transition.

Study	Country	Sample Characteristics	Aim	Results	Practical Applications
Holt and Mitchell [53]	England	*n* = 12 (*n* = 9 players; *n* = 1 youth team coach; *n* = 1 youth section director; *n* = 1 first team manager); Adults Football	Psychological aspects of the talent development.	Success in the transition to professional football often depends on psychological factors, as many young players, despite having goals and motives, lack clear pathways to achieve them.	A useful framework for understanding psychological issues that enable some talented adolescent football players to make it to professional adult football.
Van Yperen [49]	Netherlands	*n* = 65 male (14–15 year, 15–16 year, 16–17 year, and >17 year) Football	To Identifying Psychological Factors That Predict Career Success in Professional Adult Football.	Becoming successful in football is a complex and delicate process influenced by psychological, physical, social, and organizational factors, with psychological aspects such as goal commitment, coping behaviours, and social support proving decisive in differentiating youth players who reach the professional level from those who do not.	Suggestive evidence exists that both goal commitment and the tendency to engage in coping behaviours and social support seeking can change with psychosocial intervention, probably as a function of deliberate practice.
Chamorro et al. [48]	Spain	*n* = 478 (under-18 category) Football	To explore differences in young Spanish elite football players based on the importance given to getting different achievements in their future and among those players in levels of passion, motivation and basic psychological need.	A balanced engagement in multiple life domains, including education and private life, supported by passion, motivation, and the satisfaction of basic psychological needs, may better equip athletes to cope with the demands of highly competitive environments and the transition to professional football.	This idea breaks down a famous stereotype that to succeed in the transition to the elite sport level athletes must concentrate all their resources just around sport. This conception of the jump to the elite level comes closer to the holistic vision of the athlete during the athletic career.
Morris et al. [59]	UK	*n* = 5 (males) 17 and 19 years Football	To achieve experiences into the transition of young professional athletes in football.	Sources of stress within the professional football environment, including friends, family, and institutional pressures, potentially adversely affect young players’ abilities to transition successfully, while numerous sources such as friends, family, fellow professional players, coaching staff, and sport science staff provide support, and post-transition the coach becomes the most influential person in their sport, offering encouragement and emotional support, with transition experiences being influenced by the social resources they have access to and by changes in their social networks.	Moving up to senior professional football brings high motivation and self-expectations, with young players being more perfectionist and susceptible to doubts—where early support could help them manage these tendencies—while awareness of the technical and physical demands, along with perceived pressure from others, can make the step up feel less difficult, reducing anxiety and increasing confidence in their own skills.
Webb et al. [58]	England	*n* = 11 18–22 years Football	To sheds new light on the priorities and processes of talent development and education provision in English football.	There was variation in talent identification and development processes across professional football clubs, with opportunities for young players to progress to the first team depending on league level, while the transition from academy to senior squads exposed players to the emotional and perceptual-motor demands of professional competition, focusing on technical, tactical, psychological, physical, and social aspects of their development and environment.	Managers are less likely to “trust” a young player, so initial first-team opportunities, whether on loan in a lower league or within their own club, should be recognized as part of the talent development process, while developing psychosocial competencies such as resilience and providing educational support alongside football training offer benefits for adaptation to professional pressures and alternative career opportunities.
Reverberi et al. [52]	Italy	*n* = 415 *(n* = 127 league A, B; *n* = 162 league C; *n* = 128 amateur) 14–20 years Football	To understand relationship in developmental athletes according to a psychosocial approach.	The ability to stay focused on improvement and demonstrate effort is crucial for coping with difficulties and life changes, relying on social support, while the peer motivational climate plays an important role in the development of young athletes, especially in team sports.	Sport is a complex relational space where relationships play a fundamental role in athletes’ development and throughout their entire athletic career.
Silva et al. [57]	Brazil	*n* = 228 Football	To coping strategies burnout symptoms in under-20 Brazilian football players in a career transition phase and compared these variables with the occurrence of injuries and professionalization.	Being a professional athlete reflects in better performance in trainings and competitions, as well as higher exposure to pressure and stress, which can directly relate to young athletes’ negative emotions and consequently to negative coping strategies and dissatisfaction with performance, while the stress response arises from the interaction between environmental factors, such as professionalization, and personal factors, such as coping strategies, defining burnout symptoms in young athletes during career transition stages, making it essential that interventions and guidance are promoted to aid youth athletes in developing adequate strategies to cope with stress and attenuate the damages suffered during this transition.	Signing a professional contract, which reduces worries about failures, seems to favour increased confidence and motivation during training and competitions in the transition to professional football, while also possibly contributing to better responses to external performance pressure, goal planning, and mental preparation, and athletes with greater prospects for integration into the professional team may be associated with more effective use of resources to cope with stress.
Mitchell et al. [50]	England	*n* = 136 18–18 years Football	To show findings that may have implications for player experience and associated progression rates of lower categorized football academies	Successful TDEs (talent development environments) are characterized in part by their ability to provide resources for coping with future transitions, including a broad set of psychosocial skills that may facilitate successful transitions into senior environments.	This notion has been supported by football academy practitioners who cited a lack of further holistic development, isolation, and lack of coaching as barriers to successful youth-to-senior transitions.
Lundqvist et al. [54]	Qatar	*n* = 29 *(n* = 19 Europe, *n* = 5 South America, *n* = 1 Central America, *n* = 1 North America, *n* = 1 Africa, *n* = 1 Asia, *and n* = 1 Oceania. Football	To describe perspectives on demands, resources, and barriers influencing the youth-to-senior transition	The youth-to-senior transition occurs over a shorter or longer period in a complex and dynamic manner, where demands, challenges, and support needs are likely to vary over time, with awareness of physical and psychological demands, game understanding, and technical preparation perceived as transition barriers, while resiliency, motivation, high work ethic, passion for the sport, and a high level of personal responsibility, in addition to support from peers, friends, family, and coaches, are essential qualities, and moderate exposure to challenges and adversity may not necessarily affect mental health negatively but can stimulate athletic growth and resiliency if adequate support is available, with the academy and first team on the same training site easing the transition, and support programs and a club-based playing philosophy effectively promoting the transition, optimal learning environments, and enhancing psychological qualities for both performance and wellbeing, particularly resilience.	The transition from youth to senior football can be more challenging when academy and first-team players train on separate sites, as athletes cannot observe and learn from their senior peers, and factors such as biological maturity may influence the timing of the transition, while regular contact between academy players and the first team offers opportunities for stress-exposure or pressure training in ecologically valid settings, and players should not only acquire knowledge but also be exposed to specific club-based playing philosophies, understanding the roles and functions of positions within senior sport, thereby making the transition easier to manage.
McGlinchey et al. [56]	England	*n* = 4 21.6 ± 1.5 years Football	To explored players lived experiences of being released from a professional football academy	The release from professional football academies is interpreted as a process rather than a singular event, with players’ stories highlighting experiences of physical or emotional harm, feelings of helplessness, lack of control, marginalization before release, and insufficient aftercare from clubs, while players often do not seek parental support due to guilt or not wanting to burden them, despite perceiving it would be available, all of which contributes to problematic transitions out of academy football.	Psychological difficulties following release from professional football academies, including depression, anxiety, identity crisis, and loss of self-worth, along with delayed low self-esteem and confidence, coexist with potential positive psychosocial outcomes in personal development, suggesting that academies could implement pre-release programs to develop psychosocial skills and support transitions, particularly for players unlikely to become professionals or those released due to injury, with successful transitions sometimes facilitated by moving into full-time non-league football, which can enhance a player’s perceived control over their situation.
Rye et al. [55]	Norway	*n* = 10 Football	To understand the importance of adjusting stressors for players in the junior-to-senior transition in football	Levels of performance and expectations have been vital for players who successfully transition from junior to senior teams, with the most prominent competitive stress factors including pressure to perform, avoiding mistakes, and making the most of opportunities, particularly in technical skills, while players experience greater insecurity and uncertainty in performance and social interactions compared to the junior team, highlighting the importance for coaches recruiting junior players into senior football to provide support mechanisms such as senior team mentors and consultations with the senior team’s sport psychologist.	The most highlighted competitive stressors for players were the pressure to perform, particularly in avoiding mistakes and making the most of given opportunities, while they have access to performance development arenas but often lack social affiliation with senior team players, feedback from senior coaches, access to mastery-focused environments with social support, and sometimes sufficient time to prepare for senior training.
Edwards and Brannagan [51]	England	*n* = 21 (*n* = 11 professional footballers; *n* = 10 Staff members leadership) Football	To explore perceptions of players and football club staff regarding de-selection from the youth international football environment	The individual factors associated with the club-to-country transition—such as sporting performance, self-esteem, and an athletic identity tied to performance—align with those of the junior-to-senior transition, and young players wishing to remain involved in international sport must invest additional time in developing a performance-based identity.	Youth international environment acts as a similar opportunity for player development as the senior football environment, acting as a bridge between the environments of youth academy football and the perceived competitive environment of senior football.
Edwards and Brannagan [47]	England	*n* = 11 16–18 years Football	To describe experiences of youth footballers as they made their transition from club football to the England youth international football teams	High levels of social support are required to help players cope with transitions, including reflecting on performance post-camp and sharing performance data, while coaches are increasingly responsible for additional tasks beyond traditional performance duties, such as report writing and player well-being, and the most successful environments adopt an integrated, long-term development approach with a holistic view of the athlete and alignment between organizational objectives in talent development.	An increased social support structure from both teammates and coaches significantly improves the prospects of a successful senior sport transition in football, whereas a lack of high-quality social support from key stakeholders may reduce the likelihood of a successful transition.
Jordana et al. [46]	Spain	*n* = 515 14–19 years Football	To explore the psychological challenges associated with the JST in football	Irrational beliefs and extremely high standards appear to have adaptive effects on the junior-to-senior transition process, likely due to increased personal and environmental demands and challenges, while changing these beliefs during the transition could help counter the common, extreme, and irrational assumption that talented junior athletes will automatically become professional players.	Talented young football academy players generally report high satisfaction with their sports performance, yet the environments in which they develop can foster an obsession with success and failure rather than holistic growth, highlighting the need for dual-career paths that allow simultaneous academic and athletic development.
Brustio et al. [60]	Italy	*n* = 2.064: U15 (*n* = 265), U16 (*n* = 362), U17 (*n* = 416); *Primavera* (*n* = 421) and *Serie A* (*n* = 600) Football	To investigate the presence of RAE in Italian elite football players	Differences in the state of maturity within age groups negatively affect the identification of talent because relatively older players are more likely to be selected. In this sense, not only are young athletes with more advanced physical maturity given more opportunities to play, practise and train, but they may also have a better chance of becoming elite senior athletes.	The RAE limits the possibility of potentially selecting talented athletes born at the end of the reference year.
Götze and Hoppe [61]	Germany	*n* = 1763 *(n* = 1.083 male; *n* = 680 female) Football	To consider (dis)advantages in competitive sports associated with the RAE	Relatively older players were three times more likely to be recruited compared to relatively younger players; however, when analysing the conversion rate, relatively younger players were four times more likely to achieve a professional contract once they were selected for the academy.	This goal can be achieved by overcoming great challenges in training and competition, resulting in greater resilience and a strong adaptation promoted by the challenges of being involved with colleagues who are more developed from a maturational point of view and consequently in physical abilities.
Dugdale et al. [11]	Scotland	*n* = 537 Football	To predictive utility of talent identification models.	Uncertainty around early recruitment relative to successful transition to senior football.	Physical characteristics develop substantially over adolescence, and high-performance during childhood and early adolescence does not necessarily predict success as a senior professional, with anthropometric measurements such as height and mass being the least indicative of professional contract status.
Figueiredo et al. [62]	Portugal	*n* = 131.591 *(n* = 126.285 male; *n* = 5.306 female) Football *n* = 26.425 *(n* = 23.988 male; *n* = 2.437 female) Futsal	To a better understanding of the RAE in youth players	Longitudinal research into the RAE showed that male footballers born in the fourth quarter were approximately four times more likely to reach adult professional status than players in the first quarter, despite the small number of players in the fourth quarter;	This reinforces the changes associated with the transition from youth to adult professional level, which has implications for the RAE.
Bolckmans et al. [63]	Belgium	*n* = 151 15–18 years Football	To examine any possible relationship between maturity and personality constructs.	Late-maturing players tend to secure higher-value senior professional contracts in the long term, with self-regulation serving as a key personal construct in youth elite football for effective learning and potential development, while exposure to challenges requiring adaptive psychological and behavioural skills is essential for achieving excellence at senior levels, and initial physical disadvantages may ultimately contribute to multifactorial superiority once developmental differences stabilize at the elite professional stage.	Delayed benefits can be realized if late-maturing players are sustained within the talent development system, as they may develop a stronger psychological profile as senior professional players, and after selection, those born in the last quartile can sign professional contracts and benefit from increased training and competitive matches against older opponents.
Boccia et al. [64]	Italy	*n* = 1.133 *(n* = 426 U16; *n* = 570 U19; *n* = 137 Senior) Football	To explore if RAEs affect this junior-to-senior transition	Many young players were “tested” during the junior ranks, with only a few subsequently confirmed for the senior category, highlighting that youth experience is a limited indicator of senior success, and that players born earlier were often wrongly assumed to be destined for success, while those who failed to transition from juniors to seniors exhibited an even more pronounced RAE.	Selection for a youth national team is not a prerequisite for achieving senior national team call-ups, as only a few players chosen for junior national teams later join the senior national team.
Morganti et al. [65]	Italy	Study 1: *n* = 2.030 (youth national teams) Study 2: *n* = 182 (senior national team; *n* = 58 world and European championship) Football	To analyse how RAE at youth levels can impact transition at senior levels	Early born players remained overrepresented among those completing the youth-to-senior transition mainly because they were more represented at youth level, while many relatively older players who succeed at youth level may not be prepared for senior-level challenges, suggesting that a functional approach to talent development may be more appropriate for nurturing players toward senior success.	Players born in the fourth birth quartile must face greater challenges to fulfil their potential, developing more robust coping mechanisms, resilience, and motivation that help build the character needed for a successful youth-to-senior transition, while relatively younger players who enter the national system at an earlier age are also more likely to achieve football success at the senior level.

##### Physical Factors

Three main sub-themes were addressed in studies that focused on athletes’ physical characteristics: (1) maturity factors, (2) body growth, and (3) RAE (Table 1). Maturation plays a crucial role in the development of athletes, influencing how talent is perceived. Players who mature early often have temporary physical advantages, which leads to them being recognized as more talented football players. This highlights the need for careful evaluation beyond physical maturity to ensure a fair assessment of talent [63]. Maturation is the process through which individuals progress toward adulthood, involving structural and functional changes in their bodies as they transition from youth [66]. Although their chronological age may be similar, there is significant inter-individual variability in maturation status [67]. Maturation status refers to the stage of maturation at a specific point in time, representing an individual’s biological development at the moment of observation [68].

Body characteristics develop significantly during adolescence. Players who perform highly in childhood and early adolescence may not necessarily become successful professionals in adulthood [11]. About RAE, five studies compared relative age as a factor in selection or non-selection to become elite senior players during the transition phase [60,61,62,64,65]. Male footballers born in the fourth quarter are around four times more likely to reach professional status as adults than those born in the first quarter. Despite being fewer in number, players born in the fourth quarter face greater challenges in reaching their potential and this adversity helps them develop stronger coping mechanisms, promoting resilience and motivation. These characteristics are crucial for a successful transition from youth to senior level [61,62,65].

#### 3.3.2. Task Constraints

##### Type and Amount of Practice

Some studies (Table 2) have shown the importance of training type and how essential they are to deepening knowledge [30,69,70,71]. Adapting to senior competition after ten years of youth football practice was identified as necessary for all the participants, requiring greater mental and physical demands [30].

**Table 2:** Summary of studies that reported task constrains.

Study	Country	Sample Characteristics	Aim	Results	Practical Applications
Kannekens et al. [71]	Netherlands	*n* = 105 (age 16–18 years) Football	To identify possible key factors that help in predicting success over time.	Positioning and deciding is the tactical skill that best predicts the performance level in adulthood, with a correct classification of over 70% in players who are about to make the transition from youth competitions to the adult competition	Tactical expertise is a prerequisite for expert performance in sports and demonstrates the quality of a player to perform at the highest level.
Ford and Williams [70]	England	First Group: *n* = 16 (professional); Second Group: *n* = 16 (non-professional); (age 15 at the time) Football	To examine differences in the development pathways of elite youth football players in England who progressed to professional status in adulthood compared to those who did not.	After starting in football at 5 years of age, professional players in England followed the early engagement pathway throughout childhood during which they spent more time in football specific practice and play activity compared to those who did not progress to professional status in adulthood.	The development path of young players can be conditioned by the quality of practice and the path followed, whether in academies or not, may not be ideal for future performance.
Hendry and Hodges [69]	Scotland	*n* = 102 (born in 1996/1997) Football	To explore sport-specific practice on the development	Players that transitioned to adult-professional status from early youth elite levels gained early entry into a football academy, engaged in high volumes of football specific practice and play activities throughout their youth careers (defined by majority engagement in football from childhood), and participated in several sports other than football during childhood.	Athletes that successfully transition to adult professional football are best characterized by an early (majority) engagement pathway.
Swainston et al. [30]	England	*n* = 6 17–18 years Football	To describe evolving perspectives of young players experiences going through the junior-to-senior transition in professional football.	The transition from the academy to the first team is initially marked by the pressure of securing a professional contract. Contract decisions, adaptation to senior competition, barriers to transition without initial success, and social and psychological aspects of life—such as education, interpersonal relationships, and future vocation—provide unique contributions to the literature. Adapting to senior competition after ten years in youth football is identified early as a priority requiring greater mental and physical demands. Being in the first team changing room and in first-team stadiums is reported as valuable for adaptation and motivation, while the lack of first-team opportunities remains a significant barrier to successful transitions.	Players were required to adjust to training demands and a new social dynamic while learning new avenues of formal and informal support from the organization, with being on loan or playing with the U23s forming part of the process, as players focused on continuing to work toward their goals; the loan system served as the primary method for adapting to the physicality, decision-making, and style of play demands of senior football, alongside the distinct focus on winning matches and accumulating positive statistics, while the U23s offered opportunities to impress first-team staff; being comfortable with senior players and performing on the pitch helped in being accepted within the senior team, and social skills should not be overlooked, with opportunities for interaction between academy and first-team players helping to bridge the transition gap; additionally, the organization’s culture is fundamental, as initial negative perceptions—including demoralization, feelings of being ignored or unsupported, and the importance of patience and effort—play a decisive role in enabling opportunities over time.

#### 3.3.3. Environmental Constraints

##### Organization/Club Environmental and Dual Careers

Table 3 summarizes studies that combined organizational, communication, coaching and dual career, or in addition, covered other topics. A considerable number of studies (*n* = 10) are focused on understanding the main factors (e.g., competitive contexts, player development organizational structure, resources and barriers) that contribute to attain or develop expertise in the management factors. Four studies present organizational issues and the influence of strategies (e.g., communication between youth and senior team, players recruitment diversity, individual players development) in the sports context as a determining factor for success [72,73,74,75]. Six studies identify the importance that coaches play in the development of young athletes, covering technical and personal aspects, as well as communication and dual careers [76,77,78,79,80,81].

**Table 3:** Studies that addressed predominantly environmental constrains.

Study	Country	Sample Characteristics	Aim	Results	Practical Applications
Vaeyens et al. [76]	Belgium	*n* = 2.138 aged 16–39 years Football	To evaluate the benefit of the U-21 Belgium rule.	Many football teams appear either to lack the ability to develop young native players or are reticent to develop local youth talent to a level that allows under-21 players to be integrated into the first team, while reduced playing opportunities for younger football players occur because coaches are somewhat reluctant to select them for first-team matches due to their lack of match experience.	The most gifted youngsters had already been transferred to better sides and given opportunities to play in those first teams, while the reduced playing opportunities within the under-21 group in this study may stem from the ability of coaching and management personnel within clubs to effectively foster their existing young talent.
Relvas et al. [77]	Different countries (England, Portugal, Spain and Sweden)	*n* = 26 (HYD—head of youth departments); England (*n* = 6), Portugal (*n* = 5), Spain (*n* = 9), France (*n* = 2) and Sweden (*n* = 4) Adults Football	To explores the organizational structure and working practices of professional football clubs concerning young player development.	All the clubs noted that the main objective of their youth development programme was to develop players for their first team but there is an apparent gap between the first team, and the youth environments acts as an additional barrier to the player’s progression.	The lack of proximity and formal communication between the youngsters and the professional environment, regardless of the structure, causes dissatisfaction among the staff and seems to hinder the coherent progression of young players into the professional environment.
Larsen et al. [78]	Denmark	*n* = 22 (under 17 players) and the staff (club house manager; youth coaches for the under-13, under-15, under-17, and under-19 teams; sport manager; and team assistants) *n*= NR	To describe the holistic ecological approach to research in talent development in sport highlights and hot it affects an athlete in his or her athletic development.	The transition from youth to professional football often suffers from a lack of proximal role models and communication between the youth and professional environments, while coaches have a special responsibility to support athletes in this transition and athletes must be instructed on how to maintain control over their activity and investment in elite sport.	The environment can be centred on the relationship between the players and a team of coaches, assistants and directors who help the players focus on the important issues. A holistic lifestyle, dealing with dual careers (sport and school), developing the ability to work hard and be self-aware and responsible for your own training.
Larsen et al. [81]	Denmark	*n* = 22 (under 17 players) and its associated coaches; Football	To present an ecological-inspired program and intervention in a professional football.	This subsequently led to another problematic characteristic, namely that communication between coaches and players about challenges, expectations, and potential pitfalls in the youth-to-professional transition was non-existent at the club, and closely linked to the culture, the initial assessment showed that important psychosocial skills—such as managing performance and process outcomes, coping with adversity, and setting personal goals—were not taught as a natural part of training, even though they were often emphasized as very important for the within-career transition to the professional level.	Missing link between the youth and the professional players resulting in a lack of role models, and a lack of focus on the development of psychosocial skills.
Morris et al. [72]	England	*n* = 17 *(n* = 14 male; *n* = 3 female); 18 to 62 years Football	To analyse the demands, resources and barriers associated with the youth-to-senior sport transition	Coaches can provide support, and experienced sport physiologists may have a greater understanding of how to assist players with physical development compared to coaches with limited training in this area, so providing coaches with education in sport physiology and psychology can help them support athletes transitioning from youth to senior sport, alongside their motivation to help young athletes advance; organizations used staggered introductions into the senior team to allow players to assess their abilities and integrate into the squad, while educating parents on ways to support their sons, and transition outcomes were influenced by structured transition programs, with financial investment in youth setups not necessarily correlating with higher player development rates.	Knowledge of the youth-to-senior transition in sport allows practitioners to support athletes from different backgrounds and cultures appropriately, while a proactive approach to identifying factors influencing the transition and creating a list of contributing variables may yield positive outcomes; however, when players face negative responses from fans after moving up to the first team, they may struggle to succeed, highlighting areas of good practice such as staggered first-team entry, and financial investment can enable clubs to provide greater support, through sport science, education programs, and other resources, thereby enhancing youth athlete development and increasing player retention.
Aalberg and SÆTher [73]	Denmark	Head coaches for the team including head coach, chief of development and top player development coach, and all well experienced youth level coaches *n* = 6 players Football	To focused on individual development and how external factors affect athletic performance	A weak relationship between the youth department and the professional team hinders the exchange of knowledge, proximity, and fluid communication between the U19 group and the professional team, as clubs aim to “protect” senior players while motivating youngsters to compete for their place, yet fail to foster stronger links, making the gap seem large, whereas providing players with a well-coordinated environment, including coordination between the sport and collaborative educational institutions, proves crucial for enabling individual performance and for the environment’s capacity to develop successful athletes.	The focus on providing players with tools and resources both on and off the pitch, using a holistic and systematic methodology, highlights that the missing link between the youth level and the professional team may indicate that these groups do not operate under the same streamlined approach, providing a clear rationale for implementing a common philosophy and shared goals.
Hendry et al. [79]	Scotland	*n* = 102 (born in 1996/1997) Football	To consider the implications for talent development models and purported links between play and creativity.	For the transition to youth-professional, physical skills matter for selection (as do tactical and technical skills), such that concerns over selection bias towards more physically capable players in adolescence appear valid.	Coach evaluations are key determinants in future decisions about successful progression to professional youth and adult status.
Carpels et al. [74]	Different countries (England, Italy, Spain, France and Germany)	*n* = ~12.000 Football	To compare the effectiveness of this direct youth-to-senior pathway	The realistic chances of young players successfully transitioning to an elite-level first team are minimal, with some suggesting a 0.012% success rate, yet financial constraints may actually increase the success of academy graduates, as club training players (CTPs) can provide significant returns through income generation or future sales, and qualifying a player as a home-grown player (HGP) incentivizes clubs to invest in youth development; players in this category undergo continuous development and maturation appropriate to their age, and clubs should exercise patience in managing CTPs, particularly as increased match involvement does not appear to hinder club success.	The mobility of players and the internationalization of club squads allow clubs to recruit globally, bringing specific qualities they seek, and an indicator of successful academy production would be the proportion of academy graduates playing in professional senior squads, including association-trained players (ATPs) and expatriates (EXPs), regardless of origin.
Mannix et al. [80]	USA and Canada	*n* = 80 Football	To explore organisational aims and structure	Young players may undergo a ‘developing mastery phase’ that strategically provides an environment closely replicating the first team, while coaches can encourage them to increase awareness and ownership of their development through reflective practice; an increase in physical demands, noted by both coaches and transitioning players, highlights differences in training and match loads between youth and senior levels, and poor communication between the first team, reserve team, and youth academy staff across management, coaching, sport science, and medical departments may impede progression, whereas having the youth academy and first team on the same training site eases the transition by allowing young players to observe and emulate their role models.	Coaches and sports science staff in a professional club’s academy must ensure that physical development programs prepare young players for the demands of professional football, while communication among staff during the transition may be unclear or ineffective, impairing development initiatives, and although bringing youth and professional players together in a single facility can support the transition, a cultural distance still exists that creates a gap in organizational practices and communication between the youth and professional entities.
McGuigan et al. [75]	Scotland	*n* = 7 Football	To observe the factors that, including technical competence, physical process and the development environment, combine to determine the progression of young players.	The athlete’s path to elite competence in sport is rarely straightforward and typically involves challenges or obstacles, with all players who reach the first team demonstrating the ability to overcome adverse events that develop resilience and mental toughness; players who display superior physical and technical performance are more likely to succeed in the transition to senior football, as first-team coaches are less likely to trust younger, untested players, while the ideal development environment includes elite-level training, player welfare, psychological support, elite facilities for the first and B teams, an elite culture and mentality toward training and performance from all academy staff, and consideration of a wide range of player factors at both macro and micro levels, with coaches and support staff adapting team training sessions, the club philosophy, and prioritization of individual players in a team environment highlighted as key factors influencing a successful transition to the first team.	Young players are rarely exposed to adverse scenarios that allow them to develop resilience, mental toughness, and individualized development, which can hinder progression to the first team, with players possessing advanced physical and physiological capabilities more likely to advance, while a lack of specific post-academy training to meet development needs may be mitigated through loans for B team players deemed ready for first-team football, and the role of coaches or other professionals in the youth-to-first-team transition is crucial for safeguarding and supporting the development of young players within the first-team environment.

## 4. Discussion

The article aimed to systematically review and organize the available literature on the junior-to-senior transition in male football players. Our findings reveal a marked and growing scholarly interest in this critical phase of athlete development (see Section 3.3). The review highlights that a substantial body of research focuses on establishing normative benchmarks for the transition and mapping performance trajectories from early developmental stages.

The transition from youth to senior football is a complex process shaped by the interaction of psychological, physical, social, and organizational factors. Success at this stage depends not only on technical and physical proficiency but also on the ability to adapt mentally and socially to the demands of professional sport.

Quantitative studies identify key predictors of successful transitions, such as sustained soccer-specific practice, advanced tactical awareness, and psychological attributes like motivation, resilience, and goal commitment. Physical maturity also plays a role, as more mature players often access greater competitive opportunities. However, those who display strong psychosocial skills, notably self-regulation, balanced passion, and the ability to combine sport with education, tend to adapt more effectively and maintain long-term development. Qualitative research highlights that the transition is rarely linear, being affected by uncertainty, pressure, and organizational gaps. Communication and alignment between academy and senior environments are decisive: while disconnection and limited psychological support hinder progress, shared methodologies and unified club philosophies promote smoother integration. Coaches emerge as key mediators, providing emotional and practical guidance during adaptation to new professional demands.

Across studies, common themes include the importance of psychological resilience, motivation, and organizational coherence. Environments that are both challenging and supportive foster players’ confidence and coping abilities. Ultimately, effective transitions rely on holistic, individualized approaches that integrate psychological support, coordinated club structures, and authentic learning opportunities within professional settings, allowing clubs to convert potential into sustainable performance.

These insights underscore the complexity and multifaceted nature of progressing from youth to elite senior football, emphasizing the need for continued, nuanced investigation.

### 4.1. Performers Constraints

#### 4.1.1. Psychosocial Factors

##### Sources of Stress, Goals and Motivation

The limited opportunities for progression and the significant challenges associated with the junior-to-senior transition may contribute to mental health difficulties among talented youth football players [82,83]. Studies examining sources of stress during the junior-to-senior transitions consistently highlight competitive stressors, including, perform pressures, fear of making mistakes, the need to capitalise on limited opportunities, and insufficient time to prepare for senior-level training [55]. First-team experiences and opportunities should be viewed as integral components of a structured and holistic development process [58] ensuring that even those athletes who have signed professional contracts, despite being particularly vulnerable during this phase, receive appropriate support and are better equipped to employ effective coping strategies [57].

According to Morris, Tod and Eubank [59], within-career transition experiences among highly motivated athletes often generate both self-imposed and externally imposed pressure to achieve success. Given the numerous challenges faced during the junior-to-senior transition, many players fail and are subsequently released from professional football academies. This release can lead to significant psychological consequences, with players reporting experiences of depression, anxiety, identity disturbance, diminished self-worth, and reduced confidence, all of which negatively impact the quality of their transition out of full-time football [56]. Therefore, the implementation of preparatory programmes becomes essential, aiming to equip young players with psychological resilience, career adaptability, and coping strategies to deal with uncertainty and potential deselection. Such programmes could focus on mental skills training, identity management, educational guidance, and the development of alternative career plans, thereby promoting a smoother and more sustainable transition experience [52]. Football academies could develop pre-release programs aimed at preparing players for release by developing a series of psychosocial skills, which could support their transition out of football. Given the highly competitive nature of professional football and the limited opportunities for progression, it is crucial that players are encouraged to proactively prepare for alternative career pathways, thereby promoting long-term well-being and adaptability beyond football.

Goals or life aspirations that people pursue can influence their well-being, their progression within sport and the success of their careers [84]. Motivation plays a fundamental role in sports as it influences why and how athletes engage in the activities they have chosen, affecting the quality of their engagement and ultimately their performance [85]. As stated by Holt and Mitchell [53], pathway thinking includes strategic career planning and involves taking personal responsibility for development rather than only working hard for coaches. This was reminiscent of the notion of volition [86], who suggest that volition consist of knowing how to motivate oneself when other control processes fail. Volition involves working hard (especially training hard) to pursue a goal for oneself rather than uniquely appeasing one’s coaches, and it is closely related to the notion of planning careers. Hope theory [87] is based on the belief that goals are attainable, viewing hope as a positive motivational state driven by agency and pathway thinking, with goals serving as its foundation. Emerging evidence suggests that successful athletes are more likely to engage in strategic career planning, whereas their less successful counterparts often do not [53]. These differences highlight the importance of both personal and institutional support mechanisms in distinguishing elite from sub-elite athletes.

##### Social Support

Football support derived from key interpersonal relationships (e.g., coaches, parents, peers) in a sporting context has been identified as an important resource for athletes [88]. In a highly competitive environment, athletes who were able to adapt to the stressful circumstances possibly developed coping skills and social resources more frequently and more flexibly than those who did not encounter those circumstances [49]. Also, those athletes who attach importance to achievements in multiple spheres of life may become better prepared to deal with the transition to elite or professional football [13]. Some studies [50,89,90,91] reinforce the idea of developing policies among key stakeholders, ensuring the creation of supportive environments for talent development. These supportive environments include: (1) an integrated approach to player growth; (2) alignment between organizational objectives; (3) the establishment of strong social support structures; (4) a commitment to player well-being [47].

Jordana, Ramis, Chamorro, Pons, Borrueco, De Brandt and Torregrossa [46] explored the fact that many football academies are completely focused on turning talented youngsters into professional football players, overlooking that the majority won’t reach that goal. As a result, only a minority of players achieve professional status, while many others are left without adequate resources to cope with adversity during transition. However, the findings suggest that implementing proactive intervention programmes, targeting the demands, barriers, and available resources associated with transitions, could not only support the holistic development of young athletes but also enhance club outcomes in terms of reputation and long-term financial performance.

##### Commitment and Resilience

Some studies have addressed the importance of commitment and resilience in athletes. Resilience can be defined as a dynamic process of positive adaptations within a context of adversity [92,93], protecting young athletes from stressful situations or threatening scenarios [94]. In turn, sport commitment is characterized by the desire to continuously participate in a sport over time [95], maintaining adequate levels of dedication to achieve success in the progression of their careers in high performance or professional academy structures [96]. Individual factors, namely, sporting performance, self-esteem, and the athletic identity along with investing more time into the development of a performance-based identity are tied to a better performance [51] which, in turn, correlates with the influence that commitment and resilience have on a successful junior-to-senior transition [97]. Finally, Lundqvist, Gregson, Bonanno, Lolli and Di Salvo [54] referred resilience and motivation as essential player’s qualities for successful transitions, with players taking personal responsibility for their development, supporting them to stay involved in sport and better cope transitions and difficulties better [52].

#### 4.1.2. Physical Factors

##### Maturity Factors

Biological maturation can significantly influence young players’ transition to senior football. Bolckmans, Perquy, Starkes, Memmert and Helsen [63] found that early-maturing players are often perceived as more talented due to temporary physical and fitness advantages. In contrast, late-maturing players must rely more on technical skill and creativity to compensate. Interestingly, initial physical disadvantages can evolve into long-term developmental benefits, as those who remain in the system may gain multifactorial superiority. Bolckmans and colleagues also reported a reversal of maturational bias during the junior-to-senior transition, suggesting that biologically younger players may eventually outperform their earlier-maturing peers. Notably, eliminating the relative age effect in academies may unintentionally diminish the developmental advantages associated with the “less likely players” hypothesis, which posits that relatively younger athletes benefit from competing against more mature peers.

These less likely late-maturing players to succeed hypothesis posits that the junior-to-senior transition bias in elite football may favour chronologically younger players within elite youth academies [98]. To remain competitive and secure retention in academies, relatively younger and late-maturing players often must independently develop creativity alongside advanced technical, tactical, and psychological skills. Although these abilities may be less evident in childhood and early adolescence, they typically become more pronounced during late adolescence and early adulthood, as differences in age and physical maturity lessen or even reverse [99]. Late-maturing players may also benefit from prolonged childhood and adolescent stages, allowing for longer learning and perceptual-motor skill development, further enhancing their creativity as football players [100]. Importantly, the less likely players to succeed hypothesis only holds if relatively young and late-maturing players are selected and retained in youth academies. The challenging environment they navigate is key to encourage and facilitate the development of superior technical, tactical, and psychological skills [98], because these skills might only become evident as players mature and transition into higher levels of competition. These findings are supported by Ostojic and colleagues, who demonstrated that late-maturing football players are more likely to achieve success at the elite level compared to their early-maturing peers [101].

##### Physiological Adaptations

Like maturational factors, physiological adaptations in young elite footballers, assessed through a talent selection model that incorporates anthropometric, maturational, physical fitness and motor coordination characteristics, are used as predictors of future career success [102]. Dugdale, Sanders, Myers, Williams and Hunter [11] found that players who were recruited earlier from the academy showed poorer physical performance during their first years at the academy; however, the rates of development observed throughout adolescence were substantially higher for successful players, contributing to increased performance during the transition from youth to senior football. Although there were several major limitations in this study, we acknowledge the widespread use of reductionist physical qualities assessments to support and influence decision-making for the selection, deselection and progression of players in the academy system [102,103]. More importantly, numerous factors beyond isolated physical attributes influence player success and career progression. Early maturing players benefit from temporary physical advantages, which can influence selection and early performance, but these advantages often fade over time and do not guarantee senior-level success. In contrast, late-maturing players may develop superior technical, perceptual, and tactical skills, which become increasingly evident as they reach late adolescence and adulthood. Accounting for maturational status in training design, load management, and selection strategies can enhance both fairness and the effectiveness of transitions. Integrating these physical considerations with broader developmental factors ensures that talent identification and player support systems capture potential beyond immediate physical performance, ultimately facilitating a more equitable and successful progression from youth to senior football [104,105].

Thus, physical performance alone cannot determine advancement. Researchers should adopt a holistic approach that integrates a wider array of performance qualities to better identify meaningful indicators of successful junior-to-senior transitions.

##### Relative Age Effect (RAE)

Variations in maturity within age groups can hinder talent identification, as relatively older players are more frequently selected. Pressure on clubs and coaches to deliver immediate results often leads to favouring players with a short-term advantage due to their birthdate, thereby risking the exclusion of those with greater long-term potential. These studies demonstrate how selection processes are strongly influenced by RAEs, particularly benefiting players born in the first half of the year [60,61,62,64,65]. However youth experience is a limited indicator of success in the senior team [64], contrasting with other finds that indicate that relatively younger players are more likely to reach football success at senior level [65] or to achieve a professional contract [61].

The RAE has been explained based on cognitive and physical maturation. Athletes who were born earlier (relatively older athletes) in the selection year had significant advantages when compared with those who were chronologically younger (relatively younger athletes) [106]. This tendency might have reduced the chances of discovering talented athletes born later in the reference year [60] or players born earlier may be mistakenly perceived as more likely to succeed and cannot be confirmed for the senior category [64]. An RAE in adult football could be accompanied by a loss of valuable players during the youth phase of the career and limiting the pool of talented players at the adult level [61]. In Italian football national teams, Boccia, Brustio, Rinaldi, Romagnoli, Cardinale and Piacentini [64] revealed that only 9% to 15% of junior players progress to the senior national team, with date of birth significantly influencing selection, favouring those born early in the calendar year. Brustio, Lupo, Ungureanu, Frati, Rainoldi and Boccia [60] confirmed this trend in the U-15 categories but noted that the disparity diminishes with age. These findings show that promising younger players are often overshadowed by older counterparts, causing talent loss in the early stages of development, while older players often fail to transition to senior levels, resulting in talent loss later in their journey [64]. This was confirmed by Kelly et al. [107], who highlighted that players born in the fourth quarter of the year are four times more likely to reach adult professional status compared to players born in the first quarter [62]. Considering the relevance of these data, coaches and other professionals must implement strategies to support players during junior-to-senior critical transition [61].

### 4.2. Task Constraints

#### Type and Amount of Practice

Practice design plays an essential role in the development of athletes and can directly influence their performance. Practice should replicate the technical, tactical, physiological, and psychological demands of the match, incorporating the variability and complexity of formal match performance [108]. The transition to senior competition environment, normally after a decade practicing in youth football, tends to be set as a key goal, demanding for a better preparation to the new social dynamic [30]. Two studies found that players who successfully transitioned from youth elite academies to adult professional status engaged in high volumes of football-specific practice and play throughout their youth, while also participating in multiple sports during childhood, unlike those who did not reach professional levels [69,70].

Positioning and decision-making are the tactical skills that best predict performance in players transitioning from youth to adult competitions. According to Kannekens and colleagues, players with high proficiency in these skills have approximately a sevenfold greater chance of reaching the professional level compared to those with lower scores [71].

The loan system, whereby a club temporarily transfers a player to another team, serves as a key pathway for adapting to the physical, tactical, and decision-making demands of senior football [30]. It also exposes players to the intense, result-driven pressure of senior environments focused on winning matches [109] and the challenge of managing performance expectations, such as accumulating strong match statistics at their loan clubs [110]. A second pathway to prepare players for senior team competition is the establishment of U23 teams by clubs, which offer a tailored environment and additional opportunities to facilitate the transition to the senior squad [30]. Moreover, players who felt comfortable playing alongside senior teammates demonstrated a greater ability to integrate into the squad and perform well in matches [72,111]. In turn, strong performance facilitated their acceptance within the senior team [109]. For the junior-to-senior transition, players must adapt to the demands of new practice regimen and a new social professional dynamics, while at the same time learning new forms of formal and informal support from the organization to work towards their goals [30].

### 4.3. Organizational/Club Environment

#### 4.3.1. Communication and Coaching

High-level clubs typically provide players with a well-coordinated and structured environment, offering the necessary tools and resources to enable them to concentrate fully on their on-pitch performance and development [72,73]. The development environment, the club’s philosophy and the prioritisation of individual players within the context of the team were also highlighted in the literature as key factors influencing a successful transition [74,75]. The ability to align these characteristics with the requirements of senior football highlights the importance of creating development pathways that bridge the gap between youth and senior contexts [112].

Coaches play a fundamental role in the global development of athletes and can adopt more transformational training behaviours, thus promoting a greater likelihood of athletes having positive sporting experiences [81,113], namely by increasing the opportunities of young players to play in the senior team [76] or having high quality, structured, sport specific practice, that can positively contribute to the development of football skills [79].

Additionally, verbal communication plays a crucial role in ensuring clear and consistent messages among players, coaches, and staff, which can simultaneously improve team dynamics and generate a suitable environment to develop player skills and their integration into higher levels of competition [114,115]. The communication gap between youth and professional environments, whether physical or cultural, can hinder the coherent progression of young players the first senior team [77]. Even if academies that have a variety of strategies for developing players, they may fail to prepare the players for the senior team, if communication between staff is not clear and effective [80]. Young players are rarely exposed to adverse scenarios that allow them to develop resilience, mental toughness [81,116] and coping skills adapted to their individual needs [75], which may be detrimental to their progression towards first-team. Players with advanced physical and psychological capabilities are more likely to progress to the first team [117] but there tends to be a lack of specific post-academy training to meet development needs [75]. A potential solution to this problem could be to create and strengthen a better relationship between the development department and the professional team [73] and/or the role of the coach or specialized professional, challenging those involved in the physical preparation of young players to provide a training stimulus similar to the demands of senior teams, preparing players for the transition from youth to senior team [118].

#### 4.3.2. Dual Career

Understanding the career of players through the umbrella of the Holistic Athletic Career Model [119], underlines a global approach to the career development of athletes and considers that an athletic career is defined by different stages of development that integrate other life domains [120]. In their review of dual career research, [121] identified several benefits of a dual career approach, which included individual development, enhanced sports performance and, in the longer term, enhanced life satisfaction.

Research investigating elite athletes’ career pathways showed that they typically follow one of three trajectories: (1) focusing exclusively on sport; (2) combining sport and education/work while prioritizing sports-based development; (3) constructing a stable dual career pathway. These three pathways, labelled as linear, convergent, and parallel [120], are associated with different psychosocial implications of career transitions (e.g., career ending, injury), with only the stable dual career trajectory being found to safeguard athletes’ broader personal development, employability and re-integration into society after the end of their career in elite sport [122,123]. A holistic lifestyle can integrate with dual careers (sport and school/work), developing the ability in junior athletes to work hard and be self-aware and responsible for your their training [81]. Also, some athletes have a career assistance program that organizes the athletes’ environment to their advantage [78].

### 4.4. Barriers and Facilitators

Taken together, the evidence indicates that the transition from junior to senior football is shaped by a complex interplay of psychological, physical, social, and organizational factors, which can act either as barriers or facilitators of development. According to the reviewed studies, facilitators of successful progression include strong psychological skills such as resilience, self-regulation, intrinsic motivation, and goal commitment. Players capable of managing uncertainty and maintaining focus under pressure are more likely to adapt effectively to the demands of professional football. Likewise, holistic development environments characterized by consistent technical, tactical, and psychosocial support, enhance adaptability and confidence. Close coordination between academy and senior structures, involving shared methodologies, gradual exposure to first-team contexts, and coaching continuity, further supports smoother transitions. Supportive interpersonal relationships with coaches, teammates, and family, alongside dual career pathways that balance sport and education, also contribute to players’ long-term engagement and well-being.

In contrast, the literature identifies several barriers that compromise the transition process. Chief among these are organizational misalignments, including poor communication between development levels, inconsistent coaching philosophies, and limited opportunities for young players to experience competitive senior football. Such gaps disrupt developmental continuity and foster insecurity. Psychological barriers, including performance anxiety, lack of confidence, and limited coping strategies, often emerge when players face increased external pressure without adequate support. Moreover, early maturation advantages can distort selection processes, reducing developmental opportunities for late-maturing players who might possess equal or greater potential in the long term.

Overall, these findings reinforce that the success of the junior-to-senior transition depends on the alignment of individual, relational, and structural dimensions. Clubs that promote coherent organizational cultures, sustain psychological support, and progressive exposure to senior demands are better positioned to transform emerging talent into sustained professional performance.

### 4.5. Limitations

A limitation of this review is the restriction to male football and English-language publications. This inevitably excluded evidence from women’s football but ensured conceptual consistency and maintained comparability across career structures and development pathways that differ substantially between men’s and women’s football.

## 5. Conclusions

This scoping review highlights that the junior-to-senior transition in football is unpredictable and shaped by a complex interplay of physical, psychological, technical, and social factors. Despite early demonstrations of talent, many young players struggle to adapt to the higher intensity, competition, and expectations of senior football. Success at this stage requires not only physical maturity and tactical understanding but also psychological adaptability and the ability to cope with professional demands. Players must integrate into new social dynamics, working alongside older teammates and building resilience within increasingly pressurized environments.

Crucial to this process are coaches and club structures that bridge the gap between youth and professional levels. Effective coordination, shared methodologies, and consistent communication across departments facilitate smoother transitions, while fragmented systems and inconsistent feedback hinder player development. The development of psychological skills, particularly resilience, motivation, and self-regulation, emerges as essential for sustaining progress and managing setbacks.

Interpersonal support from family, friends, and teammates helps maintain well-being, while integrating education and career planning provides stability beyond sport. A dual-career approach and exposure to progressively demanding environments foster long-term growth and readiness for senior competition.

Overall, the review identifies clear barriers and facilitators in the transition process. Facilitators include strong psychosocial support, coherent organizational structures, and coaches capable of guiding both performance and personal growth. Barriers arise from lack of communication between academy and senior teams, limited opportunities for gradual adaptation, and psychological stress linked to performance pressure and uncertainty. Ultimately, successful transitions depend on aligning individual qualities with supportive environments that treat the transition as an ongoing developmental process rather than a single decisive moment.

## 6. Knowledge Gaps and Future Directions

Despite growing attention to the junior-to-senior transition in football, notable research gaps persist. Most studies are cross-sectional, offering limited insight into the dynamic and evolving nature of the transition over time. Longitudinal research is needed to examine how psychological, physical, and social factors interact throughout development. While individual attributes such as resilience, motivation, and self-regulation have been explored, less is known about how these skills are cultivated and sustained in real-world club environments. Organizational and cultural influences, such as communication between departments, coaching alignment, and club philosophy, remain underexamined, as do the perspectives of key stakeholders, including coaches, sport directors, and families. The dual-career dimension, often cited as vital for holistic athlete development, lacks football-specific empirical investigation. Furthermore, methodological imbalances between quantitative and qualitative studies limit the integration of objective performance data with lived experiences. Addressing these gaps requires global, longitudinal, and mixed method approaches that link individual development with organizational processes and socio-cultural contexts, offering a more comprehensive understanding of the barriers and facilitators shaping successful transitions to senior football.

## Data Availability

The data presented in this study are available in the present article.

## Supplementary Materials

The following supporting information can be downloaded at: https://www.mdpi.com/article/10.3390/sports13120440/s1, Supplementary Table S1: Risk of bias assessment in quantitative studies; Supplementary Table S2: Risk of bias assessment in qualitative studies.

## Abbreviations

The following abbreviations are used in this manuscript:

JST	Junior-to-senior transition
RAE	Relative age effect
