# Supplementary material for: Barriers and Facilitators in the Junior-to-Senior Transition in Male Football—A Scoping Review
Source: Sports (Basel). 2025 Dec 5;13(12):440. doi: 10.3390/sports13120440 (PMC12736883; doi:10.3390/sports13120440)
Supplement: Supplementary file 1 [file sports-13-00440-s001.zip › sports-3928900-supplementary/400_f_1_Supplementary_Material_1_20250715.docx]

| **Supplementary Table S1.** Quality criteria used to analyze the quantitative publications. | | | | | | | | | | | | | | | | | | | | | | |  |
| --- | --- | --- | --- | --- | --- | --- | --- | --- | --- | --- | --- | --- | --- | --- | --- | --- | --- | --- | --- | --- | --- | --- | --- |
| Study | Q1 | Q2 | Q3 | Q4 | Q5 | Q6 | Q7 | Q8 | Q9 | Q10 | Q11 | Q12 | Q13 | Q14 | Q15 | Q16 | Q17 | Q18 | Q19 | Q20 | Q21 | Score |  |
| McGuigan et al. (2023) | 1 | 1 | 1 | 1 | 1 | 1 | 0 | 1 | 1 | 1 | 1 | 1 | 1 | 1 | 1 | 1 | 1 | 1 | 1 | 1 | 1 | 95,2% |  |
| Edwards and Brannagan (2023b) | 1 | 1 | 1 | 1 | 1 | 1 | NA | 1 | 1 | 1 | 0 | 0 | 1 | 1 | 1 | 1 | 1 | 1 | 1 | 1 | 1 | 90% |  |
| Edwards and Brannagan (2023a) | 1 | 1 | 1 | 1 | 1 | 1 | NA | 1 | 1 | 1 | 0 | 0 | 1 | 1 | 1 | 1 | 1 | 1 | 1 | 1 | 1 | 90% |  |
| Rye et al. (2022) | 1 | 1 | 1 | 1 | 1 | 1 | NA | 1 | 1 | 1 | 0 | 0 | 1 | 1 | 1 | 1 | 1 | 1 | 1 | 1 | 1 | 90% |  |
| McGlinchey et al. (2022) | 1 | 1 | 1 | 1 | 1 | 1 | NA | 1 | 1 | 1 | 0 | 0 | NA | 1 | 1 | 1 | 1 | 1 | 1 | 1 | 1 | 90% |  |
| Webb et al. (2019) | 1 | 1 | 1 | 1 | 1 | 1 | 1 | 1 | 1 | 1 | 0 | 0 | 1 | 1 | 1 | 1 | 1 | 1 | 1 | 1 | 1 | 95,2% |  |
| Swainston et al. (2020) | 1 | 1 | 1 | 0 | 1 | 1 | NA | 1 | 1 | 1 | 0 | 0 | 1 | 1 | 1 | 1 | 1 | 1 | 1 | 1 | 1 | 85% |  |
| Morris et al. (2017) | 1 | 1 | 1 | 1 | 1 | 1 | NA | 1 | 1 | 1 | 0 | 0 | 1 | 1 | 1 | 1 | 1 | 1 | 1 | 1 | 1 | 90% |  |
| Relvas et al. (2010) | 1 | 1 | 1 | 0 | 1 | 1 | NA | NA | 1 | 1 | 0 | 0 | NA | 1 | 1 | 1 | NA | 1 | 1 | 1 | 1 | 82,3% |  |
| Holt and Mitchell (2006) | 1 | 1 | 1 | 0 | 1 | 1 | NA | NA | 1 | 1 | 0 | 0 | 1 | 1 | 1 | 1 | 1 | 1 | 1 | 1 | 1 | 84,2% |  |
| (Larsen et al., 2013) | 1 | 1 | 1 | 1 | 1 | 1 | NA | NA | 1 | 1 | 0 | 1 | 1 | 1 | 1 | 1 | 1 | 1 | 1 | 1 | 1 | 94,7% |  |
| Larsen et al. (2014) | 1 | 1 | 1 | 0 | 1 | 1 | NA | NA | 1 | 0 | 0 | 0 | 1 | 1 | 1 | 0 | NA | 1 | 1 | 1 | 1 | 72,2% |  |
| Morris et al. (2015) | 1 | 1 | 1 | 1 | 1 | 1 | 1 | 1 | 1 | 1 | 0 | 0 | 1 | 1 | 1 | 1 | 1 | 1 | 1 | 1 | 1 | 90,4% |  |
| Aalberg and SÆTher (2016) | 1 | 1 | 1 | 1 | 1 | 1 | NA | NA | 1 | 1 | 0 | 0 | 1 | 1 | 1 | 1 | 1 | 1 | 1 | 1 | 1 | 89,4% |  |
| 1=Yes; 0=No; If not applicable, assume NA | | | | | | | | | | | | | | | | | | | | | | | |
