# Supplementary material for: Barriers and Facilitators in the Junior-to-Senior Transition in Male Football—A Scoping Review
Source: Sports (Basel). 2025 Dec 5;13(12):440. doi: 10.3390/sports13120440 (PMC12736883; doi:10.3390/sports13120440)
Supplement: Supplementary file 1 [file sports-13-00440-s001.zip › sports-3928900-supplementary/400_f_2_Supplementary_Material_2_20250715.docx]

| **Supplementary Table S2.** Quality criteria used to analyze the qualitative publications. | | | | | | | | | | | | | | | | | |  |
| --- | --- | --- | --- | --- | --- | --- | --- | --- | --- | --- | --- | --- | --- | --- | --- | --- | --- | --- |
| Study | Q1 | Q2 | Q3 | Q4 | Q5 | Q6 | Q7 | Q8 | Q9 | Q10 | Q11 | Q12 | Q13 | Q14 | Q15 | Q16 | Score |  |
| Vaeyens et al. (2005) | 1 | 1 | 1 | 1 | 1 | NA | 1 | 1 | 1 | 1 | 1 | 1 | NA | 1 | 1 | 1 | 100% |  |
| Van Yperen (2009) | 1 | 1 | 1 | 1 | 1 | NA | 1 | 1 | 1 | 1 | 1 | 0 | NA | 1 | 1 | 0 | 85,7% |  |
| Kannekens et al. (2011) | 1 | 1 | 1 | 1 | 1 | NA | 1 | 1 | 1 | 1 | 1 | 1 | NA | 1 | 1 | 0 | 92,8% |  |
| Ford and Williams (2012) | 1 | 1 | 1 | 1 | 1 | NA | 1 | 0 | 1 | 1 | 1 | 0 | NA | 1 | 1 | 0 | 78,5% |  |
| Chamorro et al. (2016) | 1 | 1 | 1 | 1 | 1 | 1 | 1 | 1 | 1 | 1 | 1 | 1 | NA | 1 | 1 | 0 | 93,3% |  |
| Brustio et al. (2018) | 1 | 1 | 1 | 1 | 1 | NA | 1 | 1 | 1 | 1 | 1 | 1 | NA | 1 | 1 | 0 | 92,8% |  |
| Hendry and Hodges (2018) | 1 | 1 | 1 | 1 | 1 | 1 | 1 | 1 | 1 | 1 | 1 | 1 | NA | 1 | 1 | 0 | 92,8% |  |
| Hendry et al. (2018) | 1 | 1 | 1 | 1 | 1 | NA | 1 | 1 | 1 | 1 | 1 | 1 | NA | 1 | 1 | 1 | 100% |  |
| Götze and Hoppe (2020) | 1 | 1 | 1 | 1 | 1 | NA | 1 | 1 | 1 | 1 | 1 | 0 | NA | 1 | 1 | 1 | 92,8% |  |
| Reverberi et al. (2020) | 1 | 1 | 1 | 1 | 1 | 1 | 1 | 1 | 1 | 1 | 1 | 0 | NA | 1 | 0 | 1 | 86,6% |  |
| Carpels et al. (2021) | 1 | 1 | 1 | 1 | 1 | NA | 1 | 1 | 1 | 1 | 1 | 0 | NA | 1 | 0 | 1 | 85,7% |  |
| Silva et al. (2021) | 1 | 1 | 1 | 1 | 1 | NA | 1 | 1 | 1 | 1 | 1 | 0 | NA | 1 | 0 | 1 | 85,7% |  |
| Dugdale et al. (2021) | 1 | 1 | 1 | 1 | 1 | NA | 1 | 1 | 1 | 1 | 1 | 0 | NA | 1 | 0 | 1 | 85,7% |  |
| Figueiredo et al. (2021) | 1 | 1 | 1 | 1 | 1 | NA | 1 | 1 | 1 | 1 | 1 | 0 | NA | 1 | 0 | 1 | 85,7% |  |
| (Mitchell et al., 2021) | 1 | 1 | 1 | 1 | 1 | 1 | 1 | 1 | 1 | 1 | 1 | 0 | NA | 1 | 1 | 1 | 93,3% |  |
| Lundqvist et al. (2022) | 1 | 1 | 1 | 1 | 1 | NA | 1 | 1 | 1 | 1 | 1 | 1 | NA | 1 | 1 | 0 | 92,8% |  |
| Boccia et al. (2023) | 1 | 1 | 1 | 1 | 1 | NA | 1 | 1 | 1 | 1 | 1 | 0 | NA | 1 | 0 | 1 | 85,7% |  |
| Bolckmans et al. (2023) | 1 | 1 | 1 | 1 | 1 | NA | 1 | 1 | 1 | 1 | 1 | 0 | NA | 1 | 0 | 0 | 78,5% |  |
| Jordana et al. (2023) | 1 | 1 | 1 | 1 | 1 | NA | 1 | 1 | 1 | 1 | 1 | 0 | NA | 1 | 0 | 1 | 85,7% |  |
| Mannix et al. (2023) | 1 | 1 | 1 | 1 | 1 | NA | 1 | 1 | 1 | 1 | 1 | 0 | NA | 1 | 0 | 1 | 85,7% |  |
| Morganti et al. (2023) | 1 | 1 | 1 | 1 | 1 | NA | 1 | 1 | 1 | 1 | 1 | 1 | NA | 1 | 0 | 1 | 92,8% |  |
| 1=Yes; 0=No; If not applicable, assume NA | | | | | | | | | | | | | | | | | | |
